# Leiomyosarcoma metastatic to the cervical spine causing a C6 compression fracture: A case report

**DOI:** 10.3892/ol.2014.2132

**Published:** 2014-05-12

**Authors:** ZHENZHONG SUN, HENG WANG, HUILIN YANG, WEIMIN JIANG

**Affiliations:** 1 Department of Orthopedics, Wuxi No. 9 People’s Hospital, Wuxi, Jiangsu 214062, P.R. China; 2 Department of Orthopedics, The First Affiliated Hospital of Soochow University, Suzhou, Jiangsu 215006, P.R. China

**Keywords:** leiomyosarcoma, spine, spinal cord compression, metastasis, corpectomy, reconstruction

## Abstract

Leiomyosarcoma is a rare malignant tumor derived from smooth muscle cells, which commonly metastasizes to the lungs, liver, kidney, brain and skin. The current study presents the case of a 42-year-old male who presented with progressive neck pain and numbness of the left arm. Spinal computed tomography and magnetic resonance imaging revealed osteolytic lesions of numerous vertebrae (C2, C3, C4, C5, C6, C7, T1 and T2). With regard to the C6 vertebra, total destruction of the vertebral body resulted in vertebral collapse and subsequent spinal cord compression. The patient underwent an anterior C6 corpectomy, reconstruction with a mesh cage filled with polymethyl methacrylate (PMMA) and open PMMA infusion to C5 and C7. The surgical procedure significantly alleviated the symptoms and obtained a reliable reconstruction. The clinical follow-up examination at 13 months was uneventful with the exception of mild numbness of the left hand since the surgery. To the best of our knowledge, this is the first case of leiomyosarcoma recurrence presenting in the cervical spine, and the present study provides insight into the use of a surgical technique that has rarely been used in the cervical spine.

## Introduction

Leiomyosarcoma is a rare, malignant connective tissue tumor that originates from smooth muscle cells and accounts for ~7% of all soft-tissue sarcomas (1). Leiomyosarcoma has a poor prognosis due to its high metastatic recurrence rate and is relatively resistant to radiation and chemotherapy. In the majority of cases, the tumor most frequently arises in the uterus, gastrointestinal tract, retroperitoneum and the subcutaneous tissue of the extremities (2,3). The most commonly reported sites of metastasis from leiomyosarcoma are the lungs, liver, kidney, brain and skin. Spinal metastases of leiomyosarcoma have rarely been reported, and all cases have been located in the thoracic and lumbar spinal regions (4). The surgical treatment for spinal metastases of leiomyosarcoma may be a considered in situations involving mechanical instability and neural compression, and is regarded as palliative, with the aim of improving the patient’s quality of life.

The present study describes a case of leiomyosarcoma metastasizing to the cervical spine in a 45-year-old male with neurological deficits. The surgical technique that was used is also described. To the best of our knowledge, this is the first case of leiomyosarcoma recurrence presenting in the cervical spine. Patient provided written informed consent.

## Case report

A 45-year-old male was admitted to the First Affiliated Hospital of Soochow University (Suzhou, China) presenting with neck pain radiating into the left arm and numbness that had persisted for five months. The pain was slowly increasing in strength and was unresponsive to analgesics. The patient’s medical history included the surgical resection of a mass in the left thigh eight months prior to admittance, from which leiomyosarcoma was histologically diagnosed. Upon physical examination, palpation over the spinous process of C6 elicited severe pain, and mild hypoesthesia on the ulnar side of the left upper extremity was detected.

**Figure 1:**
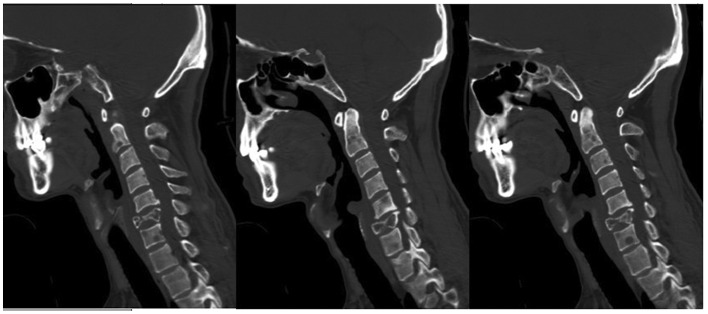
Cervical sagittal CT scans revealing complete osteolytic destruction of the C6 vertebral body and small foci in C2, C3, C4, C5, C7, T1 and T2. CT, computed tomography.

**Figure 2:**
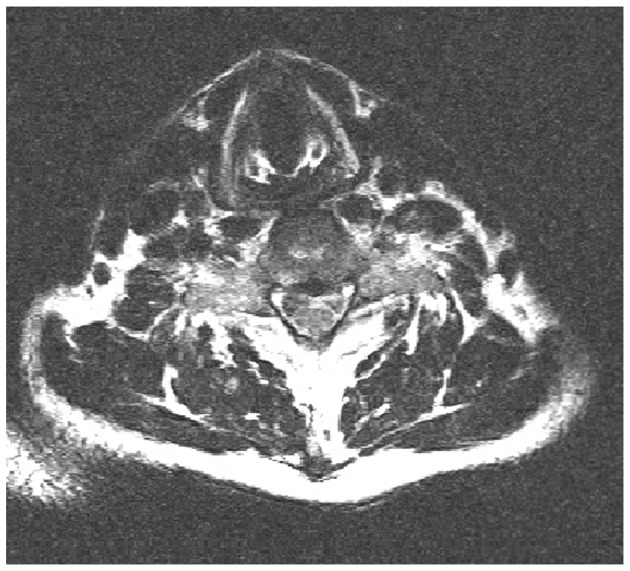
Magnetic resonance imaging demonstrating compression of the C6 vertebral body of the spinal cord.

Laboratory examinations did not reveal any abnormal findings. The plain radiographs, however, revealed a compression fracture of the C6 vertebral body. Computed tomography (CT) scans of the cervical spine (Fig. 1) revealed osteolytic lesions of numerous vertebrae (C2, C3, C4, C5, C6, C7, T1 and T2). In the case of the C6 vertebra, total destruction of the vertebral body resulted in vertebral collapse and subsequent spinal cord compression. Magnetic resonance imaging showed that the lesions were of low signal intensity on T1-weighted images and high signal intensity on T2-weighted images, and that the spinal cord at the level of the C6 spine was compressed (Fig. 2). Further CT scans of the chest also detected metastatic lesions in the lungs. Therefore, spinal metastases of leiomyosarcoma were diagnosed and decompressive surgery was selected as the therapeutic strategy.

Under general anesthesia, the patient was placed in the supine position, where the neck was slightly extended. The cervical spine was approached through a right-sided transverse skin incision. Following discectomies of C5/C6 and C6/C7, a C6 corpectomy was performed. The surgical specimen presented a grayish-white and infiltrative tumor occupying the C6 vertebral body. A frozen section was obtained intraoperatively and a subsequent diagnosis of leiomyosarcoma was formed. Following the placement of a titanium mesh cage filled with polymethyl methacrylate (PMMA) cement, the C5 and C7 vertebrae were injected with cement through a screw tract using a 5-ml syringe when the cement was at the ‘toothpaste-like’ phase. The selected screw was then inserted into the tract immediately following the cement injection.

**Figure 3:**
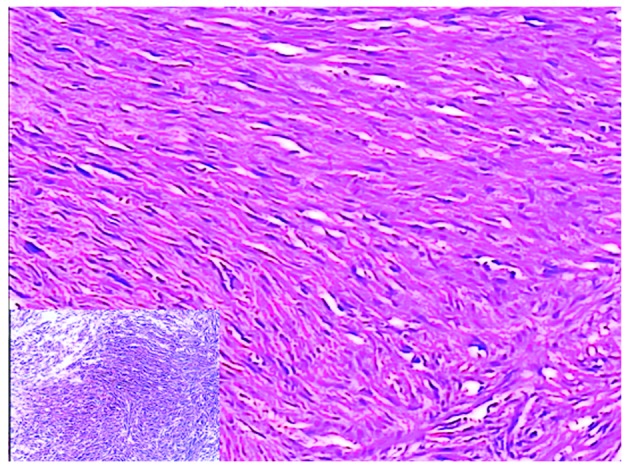
Hematoxylin and eosin staining revealing proliferation of atypical spindle cells surrounded by fibrocollagenous tissue. Immunohistochemical staining was positive for smooth muscle actin (inset).

Pathological examination of the specimen revealed a proliferation of atypical spindle cells surrounded by fibrocollagenous tissue (Fig. 3). Immunohistochemical staining was positive for desmin and smooth muscle actin (Fig. 3 inset), and negative for S-100 protein, cluster of differentiation (CD)34 and CD56. Based on the microscopic and immunochemical findings, a diagnosis of leiomyosarcoma was indicated.

**Figure 4:**
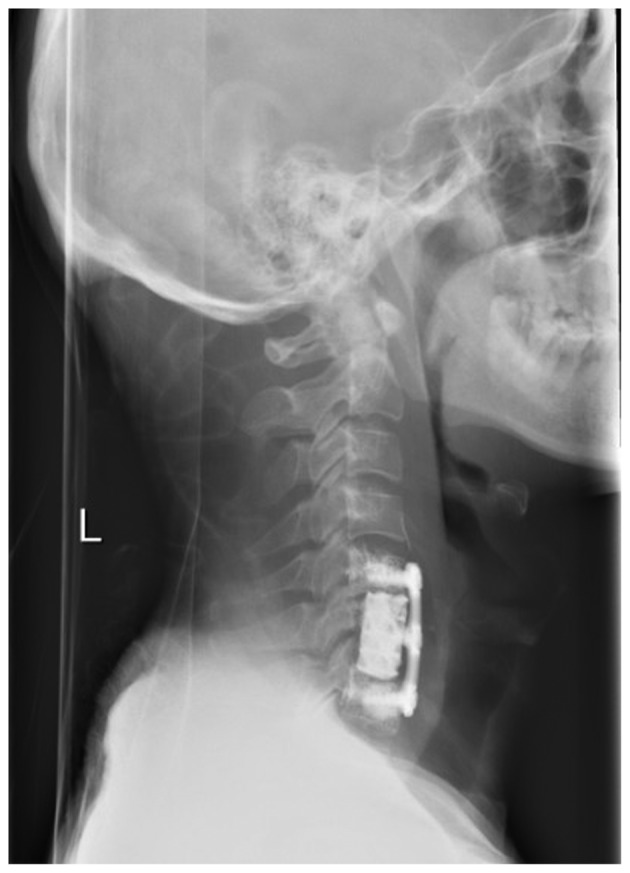
Lateral radiograph obtained 12 months after surgery revealing that no fixation failure or bone cement leakage had occurred within this time.

Post-operatively, the pain and numbness significantly decreased. The patient was transferred to the Department of Oncology for chemotherapy and radiotherapy. The chemotherapy consisted of six courses of adriamycin (25 mg/m^2^, days 1 to 3), every 28 days. Radiotherapy delivered a total radiation dose of 50 Gy in 25 fractions over five weeks. A 13-month clinical follow-up examination found that the patient had experienced only mild numbness of the left hand since the surgery. In addition, follow-up radiographs revealed that during this period, no fixation failure or bone cement leakage had occurred (Fig. 4).

## Discussion

Due to their aggressive nature and propensity for hematogenous spread, leiomyosarcomas have a strong potential for metastasis to distant sites. Osseous metastases from leiomyosarcoma are rare; the majority of cases are primary uterine leiomyosarcoma, while tumors of the stomach, vein and soft tissues have far fewer recorded incidences of metastasis to the bone (3). Despite the spine being the more common site of osseous metastasis, the involvement of the cervical spine in metastatic leiomyosarcoma has, to the best of our knowledge, never been reported previously. This may be as the cervical spine is only involved in 8–20% of metastatic spine disease cases (5), and as leiomyosarcoma metastasizing to the cervical spine may be a delayed effect and therefore of lesser clinical significance at the time of occurrence.

Destructive lesions in numerous vertebrae usually represent metastases in adult humans. In the present case, multiple cervical and thoracic vertebrae were involved by osteolytic lesions. Given the rarity of primary spinal leiomyosarcoma (6,7) and the patient’s previous history, the lesions were considered to be of a metastatic type, which was then confirmed by the pathological examination.

Microscopic examination is a reliable and important method for the confirmation that a spindle-cell sarcoma in the bone is in fact a leiomyosarcoma (3). However, distinguishing leiomyosarcomas from other aggressive spindle cell malignancies, particularly fibrosarcoma, malignant fibrous histiocytoma, malignant peripheral nerve sheath tumor and metastatic spindle cell carcinoma, is not always easy (2–4). Immunohistochemical studies are often useful in establishing the diagnosis by demonstrating the smooth muscle cell origin (3,8). Usually, leiomyosarcoma cells are positive for smooth muscle actin, weakly positive for desmin and negative for S100 protein.

The prognosis of patients with leiomyosarcoma is variable depending on the resectability and existence of metastasis. According to a previous study, patient survival times range from weeks to 13 years when leiomyosarcoma metastasizes to the spine (4). Despite numerous surgeons emphasizing that total resection of the tumor should be performed to achieve an improved prognosis (4,8,9), this was considered to be impossible in the patient in the present study. Therefore, decompressive surgery was selected as the therapeutic strategy. The surgical technique involved a C6 corpectomy with insertion of an interbody cage and anterior instrumentation. Given the poor quality of bone in the C5 and C7 vertebrae, resulting from the existence of osteolytic foci, PMMA was injected into the adjacent vertebral bodies to increase vertebral and fixation strength. It has previously been hypothesized that PMMA may have an antitumoral effect (10) and that its space occupying effect may inhibit tumor cell growth (11). It should be noted that cement augmentation of the adjacent vertebrae is not a traditional technique in the treatment of the cervical spine. However, we believe that it may have been effective in preventing fixation failure to a certain extent in the present case.

Leiomyosarcoma is known for its relative resistance to radiotherapy and chemotherapy. However, in cases where total resection of the lesion has not been established, an additional therapy, such as radiotherapy and/or chemotherapy, may be required to improve local control. In the present case, the patient was referred for radiotherapy and chemotherapy post-operatively, and in the follow-up examinations the patient exhibited only mild numbness of the left hand. However, a detailed follow-up evaluation is required.

Metastases from leiomyosarcoma should be considered in the differential diagnosis of a patient with multiple vertebrae destruction, particularly in a patient with a previous history of leiomyosarcoma. For patients with spinal metastases and neurological deficits where total resection is impossible, palliative decompression of the symptom-causing vertebrae is recommended and stability of the reconstruction should be guaranteed.

## References

[1] Russell WO, Cohen J, Enzinger F, Hajdu SI, Heise H, Martin RG, Meissner W, Miller WT, Schmitz RL, Suit HD (1977). A clinical and pathological staging system for soft tissue sarcomas. Cancer.

[2] Sanerkin NG (1979). Primary leiomyosarcoma of the bone and its comparison with fibrosarcoma. Cancer.

[3] Shapiro S (1992). Myelopathy secondary to leiomyosarcoma of the spine. Case report Spine (Phila Pa 1976).

[4] Elhammady MS, Manzano GR, Lebwohl N, Levi AD (2007). Leiomyosarcoma metastases to the spine. Case series and review of the literature. J Neurosurg Spine.

[5] Fehlings MG, David KS, Vialle L, Vialle E, Setzer M, Vrionis FD (2009). Decision making in the surgical treatment of cervical spine metastases. Spine (Phila Pa 1976).

[6] Nanassis K, Alexiadou-Rudolf C, Tsitsopoulos P (1999). Spinal manifestation of metastasizing leiomyosarcoma. Spine (Phila Pa 1976).

[7] Sucu HK, Bezircioğlu H, Rezanko T (2011). Partial spondylectomy for primary leiomyosarcoma of C2 vertebra. Spine (Phila Pa 1976).

[8] Nishida J, Kato S, Shiraishi H, Ehara S, Sato T, Okada K, Shimamura T (2002). Leiomyosarcoma of the lumbar spine: case report. Spine (Phila Pa 1976).

[9] Young CL, Wold LE, McLeod RA, Sim FH (1988). Primary leiomyosarcoma of bone. Orthopedics.

[10] Bouza C, López-Cuadrado T, Cediel P, Saz-Parkinson Z, Amate JM (2009). Balloon kyphoplasty in malignant spinal fractures: a systematic review and meta-analysis. BMC Palliat Care.

[11] Yang HL, Sun ZY, Wu GZ, Chen KW, Gu Y, Qian ZL (2011). Do vertebroplasty and kyphoplasty have an antitumoral effect?. Med Hypotheses.

